# Outpatient management of urinary tract infections by medical officers *in Nairobi, Kenya:* lack of benefit from audit and feedback on adherence to treatment guidelines

**DOI:** 10.1186/s12879-023-08567-4

**Published:** 2023-09-18

**Authors:** Florence Njeri Mbatia, James Orwa, Mary B. Adam, Gulnaz Mahomoud, Rodney D. Adam

**Affiliations:** 1 Department of Family Medicine, Aga Khan University Nairobi, Nairobi, Kenya; 2 Department of Population Health, Aga Khan University Nairobi, Nairobi, Kenya; 3 Kijabe Mission Hospital, Kijabe, Kenya; 4 Department of Pathology, Aga Khan University Nairobi, Nairobi, Kenya; 5 Department of Medicine, Aga Khan University Nairobi, Nairobi, Kenya

**Keywords:** Antimicrobial stewardship, Urinary tract infection, Audit and feedback, Diagnostic stewardship

## Abstract

**Introduction:**

Acute uncomplicated urinary tract infections are common in outpatient settings but are not treated optimally. Few studies of the outpatient use of antibiotics for specific diagnoses have been done in sub-Saharan Africa, so little is known about the prescribing patterns of medical officers in the region.

**Methods:**

Aga Khan University has 16 outpatient clinics throughout the Nairobi metro area with a medical officer specifically assigned to that clinic. A baseline assessment of evaluation and treatment of suspected UTI was performed from medical records in these clinics. Then the medical officer from each of the 16 clinics was recruited from each clinic was recruited with eight each randomized to control vs. feedback groups. Both groups were given a multimodal educational session including locally adapted UTI guidelines and emphasis on problems identified in the baseline assessment Each record was scored using a scoring system that was developed for the study according to adequacy of history, physical examination, clinical diagnosis matching recorded data, diagnostic workup and treatment. Three audits were done for both groups; baseline (audit 1), post-CME (audit 2), and a final audit, which was after feedback for the feedback group (audit 3). The primary analysis assessed overall guideline adherence in the feedback group versus the CME only group.

**Results:**

The overall scores in both groups showed significant improvement after the CME in comparison to baseline and for each group, the scores in most domains also improved. However, audit 3 showed persistence of the gains attained after the CME but no additional benefit from the feedback. Some deficiencies that persisted throughout the study included lack of workup of possible STI and excess use of non-UTI laboratory tests such as CBC, stool culture and *H. pylori* Ag. After the CME, the use of nitrofurantoin rose from only 4% to 8% and cephalosporin use increased from 49 to 67%, accompanied by a drop in quinolone use.

**Conclusion:**

The CME led to modest improvements in patient care in the categories of history taking, treatment and investigations, but feedback had no additional effect. Future studies should consider an enforcement element or a more intensive feedback approach.

**Supplementary Information:**

The online version contains supplementary material available at 10.1186/s12879-023-08567-4.

## Introduction

It is estimated that 150 million urinary tract infections occur yearly worldwide, [1] accounting for $1.6 billion in health care expenditures [2]. In the outpatient primary care setting in the USA, UTIs are second only to respiratory infections as reasons for the use of antibiotics [3] and account for 2% of annual family medicine visits [4]. They are also the most common bacterial infections in women with nearly one in three women experiencing a UTI before 24 years of age [5]. Young and sexually active women aged 18–24 years have the highest incidence of UTIs [5]. In resource-limited areas, including Kenya, UTIs are also among the most frequent infections [6, 7].

UTIs are classified into uncomplicated and complicated. Uncomplicated UTIs occur in individuals without an underlying abnormality of the genitourinary tract, and comprise the great majority of UTIs in outpatient females [4, 8]. Despite how common UTI is, the diagnoses and treatments are often suboptimal [9] leading to inappropriate treatment for the patient [10], as well as a rise in antibiotic resistance due to the misuse of antibiotics [11].

Many guidelines suggest treatment based on clinical symptoms when those symptoms are suggestive of UTI in view of data showing that the probability of cystitis is greater than 50% in women with any symptoms of urinary tract infection and greater than 90% in women who have dysuria with frequency or urgency without vaginal discharge or irritation [8, 12, 13]. In contrast, women with vaginal discharge have a high likelihood of sexually transmitted infections; thus, a vaginal examination and potential workup for a sexually transmitted infection (STI) is recommended [3]. However, urinalysis and/or urine culture are often obtained regardless of the prior probability of UTI, and their exact role in the diagnosis of acute uncomplicated UTI remains controversial [8, 14].

Unfortunately, the selection of the course of therapy for acute uncomplicated UTI has become more complex as antibiotic resistance amongst the uropathogenic strains of *E. coli* has surged globally [15]. In the Aga Khan University (AKU) population, the resistance of urinary tract pathogens to quinolones and cephalosporins is very common. For example, 21% of *E. coli* isolates from urinary tract cultures were resistant to third generation cephalosporins, 34% to quinolones and 77% to TMP/SMX, making these agents suboptimal for empiric treatment of UTI [16]*.* In contrast, only 14% of *E. coli* urinary isolates were resistant to nitrofurantoin in 2014 [16] and down to 8% in 2021 (R. Adam, unpublished data).

The currently available guidelines for acute uncomplicated UTI from Europe and North America vary due to the varying etiology and antimicrobial susceptibility patterns between these regions [17]. Recently updated guidelines of the Infectious Diseases Society of America (IDSA) and European Society for Microbiology and Infectious Diseases (ESCMID) published in 2011 stress the importance of considering the impact of resistant organisms associated with the use of antimicrobial agents [10]. These guidelines recommend nitrofurantoin for five days or fosfomycin as a single dose for acute uncomplicated UTI as the first-line option. Fluoroquinolones are reserved as a second-line option for acute uncomplicated cystitis for three days due to their adverse ecological effects [18, 19]. UTI treatment guidelines vary across geographical regions as determined by antibiotic sensitivity profiles and hospital settings [20, 21]. The guidelines we used in the present study take these recommendations into consideration and have been advised by the AKUH-N antibiotic susceptibility report for 2018 as well as the study conducted by Maina et al., [16].

Despite the availability of UTI treatment guidelines, *guideline* adherence remains inadequate in many primary care settings [9, 20, 22]. Since the availability of UTI guidelines alone does not result in improved UTI treatment outcomes, more intensive methods have been employed to enhance guideline adherence [23]. These methods include the use of audit and feedback (A&F), or the use of active or multifaceted guideline dissemination methods such as continuing medical education (CME) sessions and seminars [24, 25]. Notably, the use of guideline dissemination methods that are multifaceted or active have been shown to result in better outcomes in guideline adherence than the use of passive or single guideline dissemination methods [24]. Audit &Feedback is a multifaceted intervention but the mechanisms by which it works remains controversial in literature. Specifically; when it works, how it works and the optimum design for it to be effective. [26, 27]. Additionally, there are conflicting results on the effectiveness of audit and feedback as an intervention in influencing physician behavior [28].

In this study, we use both audit and feedback and CME as these have been shown to enhance guideline adherence in the management of acute UTIs [23, 25, 28]. This study therefore sought to determine the effect of audit and feedback and CME on adherence to locally adapted guidelines on the management of uncomplicated UTIs in female patients in the outpatient setting by medical officers. Medical officers are general practitioners who have successfully completed medical internship, are registered by the medical board, but have not undergone specialty training.

## Methods

### Study design

**Fig. 1:**
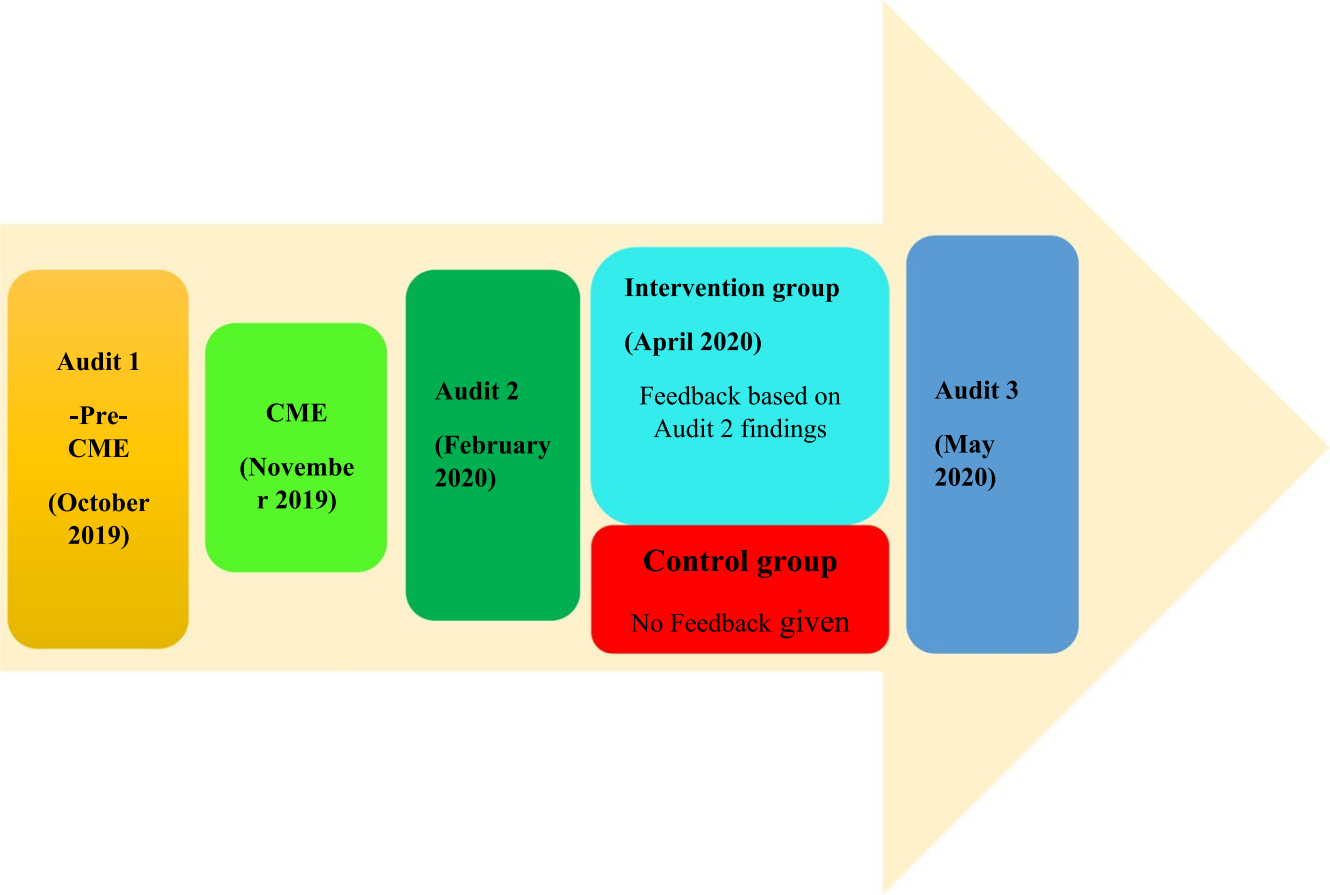
Study design and flow

We conducted a randomized control study comparing the performance of two groups of Medical Officers in their management of acute uncomplicated UTI after receiving guidelines on acute uncomplicated UTI management in form of a CME. The comparison was the effectiveness of a feedback intervention in the management of acute uncomplicated UTI. One group received feedback, while the other group did not receive feedback 4 months after the CME (Fig. 1).

### Guideline development and evidence for scoring of the five domains

In March 2019, the IDSA and ESCMID guidelines were reviewed by the Infectious Disease Specialist in the research team and acute UTI treatment guidelines were developed (Appendix 1), in the context of the AKUH susceptibility profile for outpatient urine isolates as is typically done for local guidelines. Rather than simply focusing on treatment, additional aspects of management were also included in the local guidelines as well as scoring of adherence in order to better understand the process that leads to the final outcome of treatment. The five domains (clinical metrics) included were adequacy of history, relevance of physical examination, clinical diagnosis matching the history and physical examination findings, the use of laboratory workup and treatment choice (Appendix 2 and 3). A scoring template was utilized for each of the five domains, allowing us to understand the management at a greater level of detail as is sometimes done in assessment of adherence to guidelines [29] (Appendix 3).

#### Study setting

The study was conducted in 16 Aga Khan University Hospital Nairobi (AKUH-N) outreach clinics situated within the Nairobi metropolitan area. The outreach clinics are attached to the main AKUH which is a tertiary and referral teaching hospital. The clinics offer primary care services and referrals to the main hospital for specialized medical services. The outreach clinics in Nairobi are located in peri-urban or urban shopping centers and serve patients who are mostly young, educated, formally employed and from a middle or upper middle class background. The clinics are served by medical officers, registered nurses, pharmacy technicians, laboratory technicians, radiographers and receptionist. The clinics have a pharmacy that stocks basic drugs and a laboratory to perform basic tests, including a complete blood count and urine microscopy, but all cultures are referred to the main hospital laboratory.

#### Study population

The study was conducted among permanently employed medical officers (MOs) practicing in the AKUH-N outreach clinics in the Nairobi environs. Of the 22 clinics assessed, 16 had a permanently employed MO. These 16 MOs were approached for enrollment, and all agreed to participate.

#### Randomization

Simple random allocation was applied using a random number generator to randomly allocate the 16 doctors into the two groups equally so that eight were in the intervention group and eight were in the control group. The doctors in the intervention group (feedback group) were not aware of their allocation until the time of receiving the feedback on their performance.

### Study objectives

The primary objective of the study was to determine whether there was difference between the feedback and no-feedback groups in the cumulative score covering the five domains that were scored after the CME and then after the after audit 3. Audit 3 was one to two months after the feedback session that was done in the only for the feedback group.

#### Criteria of the diagnosis for uncomplicated UTI

Patient charts were selected for non-pregnant females,14–65 years of age, and without a known structural or urological abnormality or a known immunosuppressive illness. They were considered by the study team to have acute cystitis if they presented with dysuria or frequency or urgency without vaginal discharge or irritation. Consequently, they were diagnosed as acute pyelonephritis if they had any of these aforementioned symptoms consistent with acute cystitis, and additional symptoms of nausea and/or vomiting with tachycardia and costovertebral angle tenderness.

#### Data collection

For each of the enrolled doctors, all the records of female patients from 14 to 65 years of age with a diagnosis of UTI were scored using the scoring sheet in order to know the baseline practices for workup and treatment of suspected UTI (audit 1) A second round of record review (audit 2) was performed two months after the introduction of the guidelines and provided the basis for the feedback given to the intervention group. The third record review (audit 3) occurred after feedback for the purpose of distinguishing the impact of the feedback by comparing the intervention and control groups.

### Study objectives

The primary objective was to determine whether there was a difference between the control and feedback groups in the cumulative score of all five domains from audit 3. The secondary objectives included comparing the scores of the individual components and determining for both groups whether there was an improvement after the CME in comparison to the baseline (comparing audit 1 and audit 2).

### Sample size calculation

Ad hoc power calculation was performed assuming a fixed sample size of 8 medical officers per group, effect size to be 0.8, 95% confidence level, and two sided test which yielded a study power of 85% to detect a difference on composite scores between the two groups using Mann–Whitney U test. The calculations followed the approach of Shieh and colleagues [30]. On the basis of availability of clinics and doctors, we had a predetermined samples size of 8 medical officers per group and we wanted to determine if this was sufficient to provide enough power for the study to detect the difference using Mann–Whitney U test. Since this was performed retrospectively, post hoc power analysis is the most appropriate term to use. The effect size used in the calculations was 0.8.

### The educational session (CME)

A one-hour educational session was delivered to all physicians upon enrollment into the study, either in person or by zoom.

All enrolled doctors received the AKUH newly adopted UTI treatment guidelines as an attachment on email (Appendix 1) and were reminded on a common WhatsApp group to view the attachment.

The working definition of acute UTI for the medical officers included signs and symptoms consistent with the absence of evidence of alternative diagnoses such as undifferentiated abdominal pain or vaginitis/cervicitis. The clinical diagnosis could be supported by pyuria or growth of a potential pathogen.

Key messages highlighted in the CME were:

- The 1^st^ Choice antibiotic for acute uncomplicated cystitis is nitrofurantoin.
- Patients suspected of STI and presenting with dysuria should undergo STI testing.
- Checking for costovertebral angle (CVA) tenderness in patients suspected with acute pyelonephritis is necessary.
- Documentation of the components of history and physical examination that contribute to a proper UTI diagnosis.
- Either urinalysis/urine dipstick or urine culture can be performed in acute cystitis, although empirical treatment can be given if the symptoms point to UTI as the likely diagnosis.
- Patients who fit the criteria for acute pyelonephritis should be admitted.

### The intervention (Feedback)

Individual feedback was given to the MO according to the framework of Colquhoun [26]. The doctors’ performance in each of the 5 (five) clinical performance metrics for each patient they treated was summed. The average score was calculated for each metric as well as the average score for the composite score (for all the 5 metrics) was summed up. A graph was made to capture individual doctor’s average scores and compare it to the group’s averages for each clinical metric and for total (composite) scores. The feedback was given four months after the CME session via a telephone call from author FM who was also a peer to the Medical Officers.

### Timeline

The comparative effectiveness of the feedback intervention versus the CME only intervention was evaluated at the end of the study. The study ran from the baseline period in October 2019 to May 2020 which was after the third but final audit five months after the CME. The feedback was conducted one month before this final audit.

### Data analysis

Continuous variables were expressed as median with interquartile range (IQR). The Q-Q plots and Shapiro–Wilk test showed evidence of non-normality, so non-parametric tests were used in all the analyses. The Mann–Whitney U test for non-normally distributed data was used to compare continuous variables (to compare changes from one audit to the next for both groups and to compare the two groups at each point). The test statistic, W, was the smaller of the absolute values of the positive ranks and negative ranks. This is the value we used to reject or fail to reject the null hypothesis of no difference in the median values. The difference between the medians over the three audits (first, second and third audits) were compared using Kruskal–Wallis test. Categorical variables were presented as frequency with corresponding percentages. Analysis was performed using R for windows version 4.1.1 and *p*-values < 0.05 were considered statistically significant.

### Ethical approval

Ethical approval was given by the Aga Khan Research Ethics Committee. The doctors signed consents for participation. Patient records were reviewed retrospectively with confidentiality maintained.

## Results

### Baseline characteristics of management of UTI

**Fig. 2:**
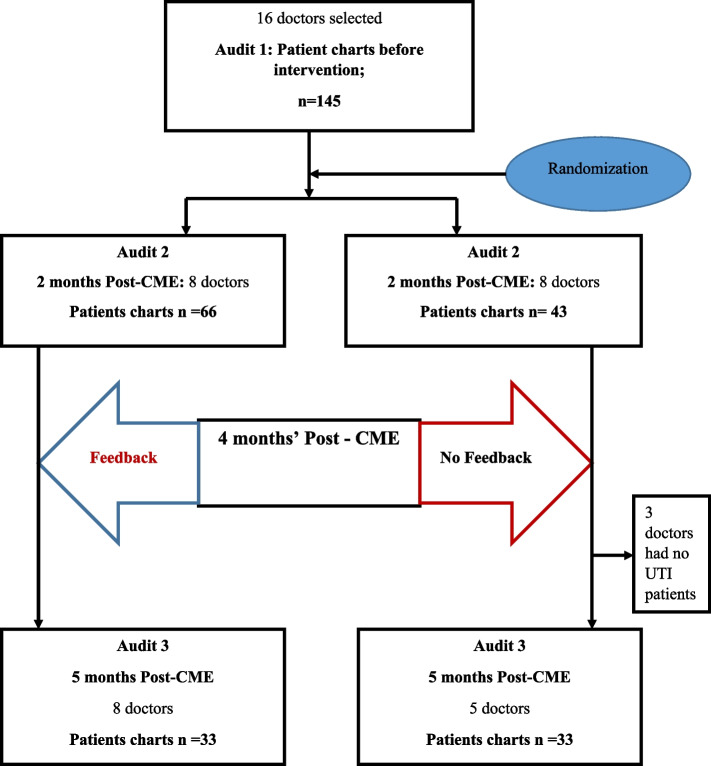
CONSORT diagram for the study

A baseline review was performed to determine the medical practice of these doctors before randomization, and 145 of those charts were for patients seen by the 16 doctors who were subsequently enrolled in the study and who fulfilled criteria for UTI as done for the chart reviews for audits 2 and 3 (Fig. 2). Among the patients diagnosed with UTI, seven (4.8%) had *history of prior* UTI within the last four weeks while two (1.4%) had *prior* UTI within the last six months. The rest of the patients either had no *prior UTI* history or no documentation of the history of UTI as reported by 134 (92.4%) and two (1.4%) of the patients respectively. Only six (4.1%) of the patients diagnosed with UTI had received antibiotics in the last three months. Penicillins (amoxicillin or ampicillin) had been used for four of the patients (Table 1).

**Table 1:** Medical officers’ overall management for UTI. for the three time periods

History	Audit 1 (Pre-CME) (*n* = 145)	Audit 2 (2 months Post-CME) (*n* = 109)	Audit 3 (5 months Post- CME, but 1 month Post-feedback) (*n* = 66)
Past history of UTI with antibiotic treatment			
Within last 4 weeks	7 (4.8)	2 (1.4)	5 (7.6)
Within the last 6 months	0	1 (0.9%)	0
No previous UTI	134 (92.4)	106 (97.2)	61 (92.4)
Not documented	2 (1.4)	0	0
Patient received antibiotics in the last 3 months for any reason			
Antibiotics	6 (4.1)	3 (2.8)	4 (6.1)
Amoxicillin or ampicillin	4 (2.75)	1 (0.92)	1 (1.5)
Cephalosporins	1 (0.7)	0	2 (3.0)
Quinolones	1 (0.7)	1 (0.92)	1 (1.5)
History items that increase (^)or decrease(#) the likelihood of patient with UTI or STI (symptoms)			
Dysuria^	82 (56.6)	59 (54.1)	52 (78.8)
Urgency^	11 (7.6)	19 (17.4)	15 (22.7)
Frequency^	42 (29.0)	34 (31.2)	18 (27.3)
Acute hematuria^	2 (1.4)	8 (7.3)	4 (6.1)
Back pain^	52 (35.9)	24 (22.0)	16 (24.2)
Suprapubic pain^#^	79 (54.5)	53 (48.6)	27 (40.9)
Fever^	10 (6.9)	10 (9.2)	4 (6.1)
Rigors^	6 (4.1)	6 (5.5)	0
Nausea^	15 (10.3)	6 (5.5)	2 (3.0)
Vomiting^	8 (5.5)	10 (9.2)	4 (6.1)
Vaginal irritation or discharge^#^	51 (35.2)	29 (26.6)	11 (16.7)
Diarrhea^#^	11 (7.6)	7 (6.4)	2 (3.0)
Upper abdominal pain^#^	19 (13.1)	9 (8.3)	4 (6.1)
***Examination*** finding ***(Signs)***			
Abdominal tenderness	46 (31.7)	71 (65.1)	20 (30.3)
Costovertebral angle tenderness	61 (42.1)	23 (21.1)	16 (24.2)
Pelvic examination (speculum or vaginal examination done)	6 (4.1)	1 (0.9)	2 (3.0)
Clinical impression			
Acute cystitis or lower UTI	144 (99.3)	109 (100.0)	65 (98.5)
Acute pyelonephritis or upper UTI	1 (0.7)	1 (0.9)	2 (3.0)
Undifferentiated abdominal pain	0	1 (0.9)	0
Pelvic inflammatory disease	0	0	0
STI	0	0	0
Additional clinical diagnosis	33 (22.8)	12 (11.0)	7 (10.6)
Diagnostic workup			
White blood cell count done	41 (28.3)	57 (52.3)	53 (80.3)
Urinalysis for microscopy done	138 (95.2)	100 (91.7)	59 (92.2)
Leucocyte count			
< 5	12 (8.3)	15 (13.8)	9 (13.6)
> 20 (numerous)	127 (87.6)	85 (78.0)	50 (75.8)
Not documented	6 (4.1)	9 (8.3)	7 (10.6)
Urinalysis dipstick done	0	2 (1.8)	5 (7.6)
Urine culture done	5 (3.4)	1 (0.9)	2 (3.0)
PCR for Neisseria gonorrhoea or Chlamydia trachomatis done	0	1 (0.9)	0
Blood culture done			
Any non-UTI laboratory tests done	20 (13.8)	4 (3.7)	5 (7.6)
Any non-UTI imaging tests done	2 (1.5)	4 (2.8)	1 (3.6)
Treatment			
Patients started on antibiotics	144 (99.3)	108 (99.1)	64 (97.0)
Type of antibiotics prescribed			
Amoxicillin or Ampicillin	2	0	1
Amoxicillin- Clavulanic Acid	4	2	0
Cephalosporins	70	53	43
Fluoroquinolones	61	43	14
Nitrofurantoin	5	10	5
Others	2	0	1
Non antibiotic treatment prescribed			
Analgesia	64 (52.0)	53 (52.5)	41 (66.1)
H2 receptor antagonists	12 (9.8)	5 (5.0)	0
Urinary alkalizers	33 (26.8)	19 (18.8)	11 (17.70)
Antacids	10 (8.1)	4 (4.0)	0
Antispasmodics (e.g. scopolamine)	23 (18.7)	14 (13.9)	10 (16.1)
Antimotility agents (e.g. domperidone)	0	2 (2.0)	0
Anti-emetic agents	5 (4.1)	1 (1.0)	2 (3.2)
Vaginal douching agents	5 (4.1)	10 (9.9)	1 (1.6)
Others	23 (18.7)	12 (11.9)	9 (14.5)
Patient referred for admission	3 (2.1)	2 (1.8)	1 (1.5)

The most common presenting symptoms were dysuria (56.6%), suprapubic pain (54.5%), back pain (35.9%) and history of vaginal irritation or discharge (35.2%) (Table 1). The rest of the presenting symptoms are as shown in Table 1. Abdominal tenderness (46; 31.7%) and costovertebral tenderness (61; 42.1%) were the most commonly documented physical examination findings (Table 1). Pelvic examination was rarely done (6; 4.1%), although (51; 35.2%) had vaginal complaints.

Urine microscopy was the most frequently conducted test (138; 95.2%), but urine dipstick and culture were rarely performed (3.4%). Blood cultures were not performed for any patient, non-UTI laboratory tests were performed for 20 patients (13.8%), while non-UTI imaging tests were performed only for two patients. Stool for microscopy (17) and *H. pylori* Ag (11) were the most frequently performed non-UTI tests. In addition, CBC was considered inappropriate in the absence of systemic symptoms [3], but was done in 28.3%. Other investigations that are non-UTI related are presented in Table 1.

### Treatment prescribed

A total of 144 (99.3%) of the patients with a diagnosis of UTI were prescribed antibiotics (Table 1). Cephalosporins and fluoroquinolones were the commonest antibiotics prescribed as documented for 49% and 42% of the patients, respectively. Relatively few patients received nitrofurantoin, amoxicillin or ampicillin and amoxicillin clavulanic acid (Table 1).

### Impact of CME and feedback

Audit 1 (baseline) showed a slightly higher score for the feedback group in the treatment domain, but all other domains and the total score were similar (Table 2). There was no difference between the two groups after audit 2 and surprisingly, the control group had a higher score after audit 3 resulting from better performance in physical examination and diagnostic approach domains.

**Table 2:** Median (Interquartile ranges) for the five clinical domains and the total scores for the two arms for the first, second and third audits

Audit Period	Audit 1 (*N* = 145) Baseline			Audit 2 (*N* = 109) After CME			Audit 3 *N* = 66 After feedback		
	**Feedback Arm**	**Control Arm**	***p*-value**	**Feedback Arm**	**Control Arm**	***p*-value**	**Feedback Arm**	**Control Arm**	***p*- value**
**History** **(Max 20)**	16 (4–19)	16 (4–18)	0.91	20 (20–20)	20 (20–20)	0.801	20 (20–20)	20 (20–20)	0.126
**Physical Examination** **(Max 10)**	6_ (5–7)	7 (4.5–7.5)	0.55	10 (7–10)	10 (7–10)	0.660	7 (7–10)	10 (7–10)	0.029
**Clinical Diagnosis** **(Max 10)**	5 (5–5)	5 (5–5)	0.44	5 (5–10)	5 (5–10)	0.973	5 (5–10)	5 (5–5)	0.639
**Diagnostic Approach** **(Max 30)**	20 (20–25)	20 (20–25)	0.19	25 (20–30)	25 (20–30)	0.844	25 (20–25)	25 (25–30)	0.003
**Treatment** **(Max 30))**	15 (15–17.5)	15 (10–15)	0.03	15 (15–15)	15 (5–20)	0.024	20 (15–20)	20 (15–20)	0.113
**Total score** **(Max 100)**	63 (54.5–70)	62 (53–69)	0.61	77 (67–85)	76 (67–85)	0.324	75 (67–80)	80 (75–85	0.008

Significant difference between the two groups shown in bold; data presented in median (IQR)

Comparisons of both groups over time indicated significant improvements in the overall scores of both groups between audits 1 and 2 (Table 3). The feedback group improved significantly in the history, physical examination and treatment scores while the control group improved in the in four of the five domains.

**Table 3:** Changes over time on adherence to guidelines from the first audit to the third audit

Category	Feedback group (% score)			*P*-value		Control group (% score)			*P* -value	
	Audit 1	Audit 2	Audit 3	Audit 1 vs Audit 2	Audit 2 vs Audit 3	Audit 1	Audit 2	Audit 3	Audit 1 vs Audit 2	Audit 2 vs Audit 3
History	62.0%	99.7	95.5	< 0.001	0.025	64.2%	99.8	99.4	< 0.001	0.414
Physical Examination	64.8%	89.3	80.9	< 0.001	0.041	65.2%	88.4	90.9	< 0.001	0.465
Clinical Diagnosis	53.0%	60.2	62.1	0.09	0.781	58.6%	61.6	59.1	0.33	0.725
Diagnostic Approach	73.7%	80.6	74.2	0.004	0.115	69.8%	79.1	86.4	0.004	0.071
Treatment	53.1%	47.3	59.1	0.38	0.006	49.2%	58.5	63.1	0.003	0.197
Total score	62.2%	73.1	73.2	< 0.001	0.961	60.9%	76.3	79.7	< 0.001	0.132

Audit 1 Baseline; Audit 2 two months post-CME; Audit 3 five months post-CME and two months after feedback for the feedback group

### Specific changes over time

**Fig. 3:**
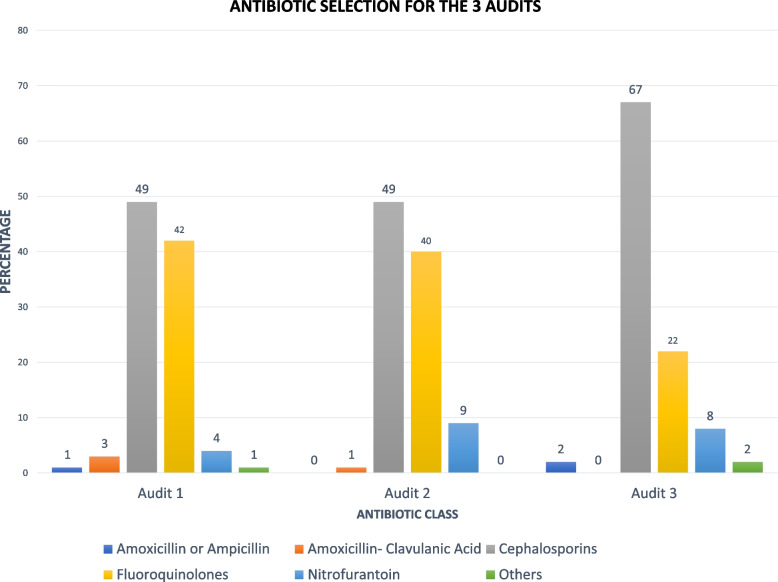
Antibiotics started following the UTI diagnosis. The percentages are out of the total number of patients for each period as follows: Pre-CME *n* = 145 Post-CME *n* = 109 and Post feedback *n* = 66

In addition to the total scores, it is informative to consider the individual changes that occurred over time in diagnostic workup and treatment. Both the feedback and control group improved substantially after the CME in comparison to baseline in the overall score with the most notable improvements coming in documentation of history and physical exam as well as the use of diagnostic testing. It is notable that the control group also improved their treatment score while the feedback group (which was yet to received feedback) had an insignificant trend toward a worse score in the treatment domain. One concerning change that occurred over time was ordering a CBC, which went from 28% pre-CME (audit 1) to 52% post-CME (audit 2), and 80% after audit 3 (postfeedback) (Table 1). There was a change in treatment that also occurred over time in both groups. Despite strongly encouraging the use of nitrofurantoin, its use went only from 2 to 8% (Fig. 3). At the same time, cephalosporin use went from 40 to 67% while the quinolone use dropped from 34 to 21% (Table 1).

## Discussion

This study is one of the few in sub-Saharan Africa to study the effect of A&F on physician adherence to UTI guidelines for doctors trained at the medical officer level (internship but no specialty residence training).

### Baseline

The baseline review of medical records yielded useful insights into the workup and treatment of UTIs in outpatient clinics throughout the Nairobi metro area. These observations then informed the areas of emphasis for the CME. A full evaluation for suspected STI was emphasized on the basis of how commonly vaginal complaints were found, with vaginal irritation/discharge found in over 35%. This requires further workup because of the significant possibility of having a sexually transmitted infection rather than UTI [3, 31, 32]. The appropriate workup includes a pelvic examination and testing for *Chlamydia trachomatis* and *Neisseria gonorrhoeae*. Although a urine culture is not necessary for evaluation and treatment of a UTI when the presentation is more typical, this is a setting where it is recommended. At baseline, only 12% of those with vaginal symptoms had a urinary culture and/or workup for STI.

An additional area of emphasis was the choice of antimicrobial agent. Some guidelines list nitrofurantoin as the empiric treatment of choice because of up to 94% and bacteriological cure rates of up to 92%, as well as lower rates of side effects in comparison to other antibiotics [33]. The importance is even greater in the local setting of much higher resistance rates for cephalosporins and quinolones than for nitrofurantoin. Published data from 2014 indicated that only 66% of *E. coli* and 76% of *K. pneumoniae* (the two most common urine isolates) were susceptible to quinolones and 74% and 62%, respectively were susceptible to cefuroxime, a second generation cephalosporin. Quinolones are particularly discouraged in view of emerging toxicities, including *C. difficile* colitis and tendinopathies [8, 34]. One explanation that the providers gave during the interactive CME and /feedback sessions was that patients often reported undesirable side effects related to nitrofurantoin such as nausea and requested an alternative medication.

### What changes were seen after feedback?

Both the control group and the feedback group demonstrated improvement from the baseline to the third audit (Table3). However, in contrast to expectations, most of the improvement was from the baseline (audit 1) to the post-CME time (audit 2). there was no improvement in the scores after the feedback. In fact, the control arm performed better overall than the feedback arm when comparing the results of the baseline and final audits. Even after audit 2, which was after CME but before feedback, the control performed better for treatment than the feedback arm. This suggest that the control arm was different at the baseline in terms of the response of the physicians to educational approaches.

Thus, the remainder of the discussion will focus on the change over time in both groups.

Since the baseline data had suggested the importance of workup for possible STIs, it was emphasized in the CME and improvement was expected. Unfortunately, this did not improve after the CME or feedback. After the CME, 27% had documented; yet only 1% had a vaginal examination performed, 1% had a urine culture done, and 1% had workup for a sexually transmitted infection. Thus, it is likely that many sexually transmitted infections are missed in the outpatient setting.

The use of diagnostic tests that are not indicated have the potential of increasing inappropriate antimicrobial use and are the focus of diagnostic stewardship interventions. One of the most common of those tests is the CBC which is commonly used in the local setting as the determinant for starting antibiotics even in the absence of indications for antimicrobial therapy (unpublished observations). Thus, it is of particular concern that the use of the CBC actually went up over time and was ordered in over 80% of the patients from the final audit. Also notable, was *H. pylori* Ag, which is commonly ordered in this setting for nonspecific abdominal pain (unpublished observations) and has the potential to promote the overuse of antibiotics.

One of the other goals of the CME and feedback was to increase the use of nitrofurantoin for UTI. but the use went up only from 2 to 8%. However, there was a notable decrease in the use of the quinolones and a compensatory increase in cephalosporin use. This can be considered a favorable although not optimal change in view of the risks for *Clostridioides difficile* disease, altered microbiota and tendinopathy associated with the fluoroquinolones. Further understanding of the reluctance to use nitrofurantoin requires further investigation to explore and quantitate the reasons.

### Strengths and limitations

One strength for this study is that feedback was given by a peer which has been shown to have positive effects as opposed to if feedback is given by a senior or a supervisor. Another strength of the study was the contribution to the current approach to management of suspected UTI by medical officers and the identification of issues that can be targeted in further interventions. The study was limited by a small number of physicians, meaning that small effects may have been missed and would have affected the generalizability of the results and its applications to a larger setting. However, it is unlikely major improvements were missed, especially in view of the small improvements in individual components such as the use of nitrofurantoin. Secondly, the small patient volumes experienced due to the COVID 19 pandemic limited the number of available charts for review. Thirdly, we may have delivered an insufficient dose (a single feedback session) of the intervention to effect practice change. *Fourthly, there was lack of data on the characteristics of the MOs, thus the years of MOs experience was not noted and may have affected the results as the study was dependent on documentation. It is anticipated that years of physician experience is negatively correlated with documentation *[35]*, as physicians who have been longer in practice may document less.*

## Conclusions

This study has demonstrated the challenges in persuading physicians to use nitrofurantoin, which is the drug of choice, not only in Kenya but globally. It also informs the need for other interventions that focus on the diagnostic side, such as STI evaluation and limiting the use of tests that do not aid in the diagnosis of UTI, such as CBC, stool microscopy and *H. pylori* Ag.

### Supplementary Information

**Additional file 1: Appendix 1. **Guidelines for diagnosis and treatment of acute UTI in women. **Appendix 2.** UTI Scoring sheet. **Appendix 3.** Scoring criteria on the Medical Officers’ adherence to locally adopted Acute Uncomplicated UTI management guidelines.

## Data Availability

Detailed data relevant to the manuscript will be provided on request to the corresponding author (RDA).
