# Supplementary material for: Outpatient management of urinary tract infections by medical officers in Nairobi, Kenya: lack of benefit from audit and feedback on adherence to treatment guidelines
Source: BMC Infect Dis. 2023 Sep 18;23:608. doi: 10.1186/s12879-023-08567-4 (PMC10506338; doi:10.1186/s12879-023-08567-4)
Supplement: Supplementary file 1 — Additional file 1: Appendix 1. Guidelines for diagnosis and treatment of acute UTI in women. Appendix 2. UTI Scoring sheet. Appendix 3. Scoring criteria on the Medical Officers’ adherence to locally adopted Acute Uncomplicated UTI management guidelines. [file 12879_2023_8567_MOESM1_ESM.docx]

**APPENDICES**

**Appendix 1: Guidelines for diagnosis and treatment of acute UTI in women**

**Introduction**

Symptoms compatible with UTI provide the hallmark for considering the diagnosis of acute UTI. Pregnancy is the only situation where there is value in treating asymptomatic UTIs. Thus, a history must focus on the symptoms that are associated with UTI. It is also important to identify symptoms that are not associated with UTI and that may point to alternative diagnoses.

**History**

Symptoms strongly associated with UTI ([Bent et al., 2002](#_ENREF_2); [Medina-Bombardó and Jover-Palmer, 2011](#_ENREF_27)).

Dysuria (especially when dysuria and urgency are both present)

Urgency

Frequency

Acute hematuria, especially with dysuria

Items weakly predictive of UTI include

Back pain

History of UTI

Factors negatively associated with UTI

Suprapubic pain

Fever (however, fever in someone with a UTI may indicate pyelonephritis)

Vaginal irritation or discharge (these symptoms are more suggestive of sexually transmitted infections (STIs) and should prompt consideration of these diagnoses. Pelvic examination should be performed in these patients.

Other important components of the history

Pregnancy/LMP

GI symptoms that may point to an alternative diagnosis, including gastroenteritis, cholecystitis, etc.

Sexual history may point to a risk factor for recurrent UTI but is more likely to point to risk factors for STIs

**Physical examination**

There are no components of the physical examination that are highly predictive of UTI. Thus, the major role is to rule out alternative diagnoses such as gastroenteritis or STI. In addition, for patients with UTI, the examination may indicate evidence of pyelonephritis (upper UTI) that will require a different approach to diagnosis and treatment.

Address abnormal vital signs

Fever may indicate pyelonephritis but more likely will point to an alternative diagnosis. Hypotension or tachycardia is not sensitive or specific for the diagnosis of UTI but will help to determine the level of treatment required.

The abdominal examination is required for the diagnosis of non-UTI diagnoses and if the abdominal exam is abnormal, would point to one of these diagnoses.

CVA tenderness, especially when unilateral is suggestive of pyelonephritis in a patient who has other signs and symptoms suggestive of UTI.

Clinical diagnosis (Categorize the illness as follows, one or more)

Acute cystitis (lower UTI)

Pyelonephritis

STI (urethritis, vaginitis)

Undifferentiated abdominal pain

Pregnancy

PID

**Diagnostic approach**

Urine culture and microscopy – Urine culture is the gold standard for diagnosis in people presenting with symptoms that are suggestive of UTI. The identification of a single organism known to be a urinary tract pathogen (e.g. *E. coli*, *Klebsiella pneumoniae*, *Staphylococcus saprophyticus*) is considered a positive result. Usually there will be 10^5^ organisms per ml, but as low as 10^3^ may be considered positive. Pyuria is usually present. More than one organism usually represents contamination at the time of collection. Most published guidelines do not recommend culture for an initial case of uncomplicated UTI. However, those guidelines are based on settings where resistance rates are low. In the current setting, there are no data to indicate whether cultures should be done routinely. Therefore, this guideline does not make a recommendation on whether a urine culture should be done routinely. However, it should be done if there is evidence for pyelonephritis.

Consider urine dipstick – However, the presence of pyuria on the urine dipstick is neither sensitive nor specific enough to reliably diagnose UTI ([Burd and Kehl, 2011](#_ENREF_3); [Oyaert et al., 2018](#_ENREF_28)). Rather, the presence of any two of (dysuria, urgency, frequency) and absence of vaginal discharge are more accurate than a dipstick urine ([Bent et al., 2002](#_ENREF_2)) .Pyuria is commonly seen in acute febrile illness and/or dehydration, so the presence of pyuria by dipstick or microscopy does not necessarily indicate UTI. In addition, pyuria (as well as positive urine culture) is of minimal significance in patients with renal failure.

In summary, laboratory testing is indicated primarily for determining the etiologic agent of UTI in patients with more severe infection or for determining complications of their illness. In addition, laboratory testing may be indicated for the diagnosis of other illnesses that must be distinguished from UTI.

- Urine culture if question about diagnosis or suspected pyelonephritis
- CTNG if suspected STI (depending on cost coverage)
- Blood culture if suspected pyelonephritis
- Other nonspecific lab is indicated only if diagnosis is unclear or if there is systemic illness

**Treatment** (based on diagnosis)

The empiric treatment regimen (treatment in the absence of culture data) is based on the local prevalence of various organisms associated with UTI and the resistance levels of those organisms. At AKUHN, the causes of UTI are as follows, based on a review of laboratory data (without clinical correlation) ([Maina et al., 2016](#_ENREF_25)).

Escherichia coli (76%)

Klebsiella pneumoniae (11%)

Staphylococcus saprophyticus (2%)

Enterococcus spp. (2%)

Proteus spp. (2%)

For *E. coli*, the susceptibilities to amoxicillin (20%) and TMP/SMX (24%) were very low, making these drugs unsuitable for empiric therapy. The susceptibilities for quinolones (66%), nitrofurantoin (86%) and third generation cephalosporins (80%) are significantly better, although all have significant resistance rates. For *K. pneumoniae*, the quinolone (76%) and third generation cephalosporins (65%) are also suboptimal and nitrofurantoin is ineffective. It should be noted that the actual susceptibility rates may be higher since the cultures from the laboratory may disproportionately be done for treatment failure and treatment failure. In view of the local susceptibilities and other published guidelines, nitrofurantoin is recommended as the first choice for lower UTI, with quinolones and third generation cephalosporins as alternatives. Nitrofurantoin is also considered a treatment of choice in published guidelines ([Gupta et al., 2011](#_ENREF_15)) and in AKUHN guidelines.

Lower UTI

Nitrofurantoin

Pyelonephritis

Supportive treatment

Most patients with a clinical presentation suggestive of pyelonephritis should be admitted to the hospital.

Patients presenting with febrile illness, especially with tachycardia or hypotension may have alternative diagnoses that must be identified. If they do have pyelonephritis, blood and urine cultures are mandatory so that therapy can be adjusted in case of resistant organisms. Be sure to collect a urine sample prior to antibiotic therapy even if in-out catheterization is required.

Antimicrobial therapy

Ceftriaxone or gentamicin are the treatments of choice for patients with pyelonephritis.

In case of septic shock (by objective criteria) or a prior history of a resistant organism from a urinary tract infection, consider a carbapenem while awaiting culture results

**Appendix 2: UTI Scoring sheet**

**History (20 points; 2 for each category addressed; need 10 for full score)**

___ Dysuria

___ Urgency

___ Frequency

___ Acute haematuria

___ Back pain

___ History of UTI

___ Suprapubic pain

___ Fever

___ Vaginal irritation or discharge

___ Pregnancy/LMP (give score for age 50 or greater)

___ GI symptoms

___ Sexual history

**Physical examination (10 points; mark each as A or I; appropriate or inappropriate)**

___ Address abnormal vital signs (fever, hypotension or tachycardia) (4)

___ The abdominal examination including CVA tenderness (3)

___ Vaginal exam if indicated (3)

**Clinical diagnosis** **(one or more) (10 points for correct, 5 for partial)**

___ Acute cystitis (lower UTI)

___ Pyelonephritis

___ STI (urethritis, vaginitis)

___ Undifferentiated abdominal pain

___ Pregnancy

___ PID

**Diagnostic approach** **(30 points; mark each as A or I; 5 points for each A)**

___ Urine microscopy and/or culture (must clarify the role of culture) (5)

___ CTNG if suspected STI (5)

___ Blood culture if suspected pyelonephritis (5)

___ Other nonspecific lab only if unclear diagnosis or systemic illness (5)

___ Unnecessary lab (1-2 tests) (5)

___ Unnecessary lab (=> 3 tests) (5)

**Correct Treatment based on diagnosis; 30 points; mark each as A or I)**

**Diagnosis is determined by what is listed by the treating physician unless the history and exam do not fit the diagnosis.**

___ Lower UTI (nitrofurantoin 5 days)

___ Correct drug (20)

___ First line drug (20)

___ Second line drug (10)

___ Correct duration (10)

___ Pyelonephritis

___ Correct drug (20)

___ Correct supportive treatment (e.g. hospitalization) (10)

___ Not UTI (30)

___ Appropriate treatment for diagnosed condition (usually no antibiotics) (30)

___ Partially correct treatment (15)

**Appendix 3: Scoring criteria on the Medical Officers’ adherence to locally adopted Acute Uncomplicated UTI management guidelines**

| **CLINICAL METRICS** | **MAXIMUM SCORES POSSIBLE** | **CRITERIA FOR MAXIMUM SCORING** | **DOMAINS** | **SCORES** |
| --- | --- | --- | --- | --- |
| **HISTORY** | 20 | Any 10 of these domains documented | Dysuria | 2 |
|  |  |  | Urgency | 2 |
|  |  |  | Frequency | 2 |
|  |  |  | Back pain | 2 |
|  |  |  | History of UTI | 2 |
|  |  |  | Suprapubic pain | 2 |
|  |  |  | Fever | 2 |
|  |  |  | Vaginal irritation or discharge | 2 |
|  |  |  | Pregnancy/LMP (give score for age 50 or greater) | 2 |
|  |  |  | GI symptoms | 2 |
|  |  |  | Sexual history | 2 |
| **PHYSICAL EXAM** | 10 | All these three domains documented | Vital signs   - Blood pressure - Heart rate | 4 |
|  | | | Abdominal exam performed with CVA tenderness | 3 |
|  |  |  | Vaginal exam if indicated | 3 |
| **CLINICAL DIAGNOSIS** | 10 | 10 points for correct clinical diagnosis, 5 for partial | Acute cystitis (lower UTI) | 10 |
|  | | | Pyelonephritis (Upper UTI) | 10 |
|  |  |  | STI (urethritis, vaginitis) | 10 |
|  |  |  | Undifferentiated abdominal pain | 10 |
|  | | | Pregnancy | 10 |
|  |  |  | PID | 10 |
| **DIAGNOSTIC APPROACH (INVESTIGATIONS)** | **30** |  | Urine microscopy and/or culture (must clarify the role of culture) | 5 |
|  | | | CTNG if suspected STI | 5 |
|  |  |  | Blood culture if suspected pyelonephritis | 5 |
|  |  |  | Other nonspecific lab only if unclear diagnosis or systemic illness | 5 |
|  |  |  | Unnecessary lab (1-2 tests) | - 5 |
|  |  |  | Unnecessary lab (> 3 tests) | - 5 |
| **TREATMENT** | **30** | Correct 1^st^ line antibiotic and duration | Lower UTI (Cystitis) (nitrofurantoin given for 5 days) | |
|  | | | - Correct drug | 20 |
|  |  |  | - First line drug | 20 |
|  |  |  | - Second line drug | 10 |
|  |  |  | - Correct duration | 10 |
|  | | | Pyelonephritis (Upper UTI), 1^st^ line is ceftriaxone/gentamicin | |
|  |  |  | - Correct drug | 20 |
|  |  |  | - Correct supportive treatment (e.g. hospitalization) | 10 |
|  | | | Not UTI | |
|  |  |  | Appropriate treatment for diagnosed condition (usually no antibiotics) | 30 |
|  | | | Partially correct treatment | 15 |
| **TOTAL MAXIMUM SCORES** | **100** |  | | |
